# Intracranial functional near-infrared spectroscopy: an animal feasibility study

**DOI:** 10.3389/fmedt.2025.1692573

**Published:** 2025-11-27

**Authors:** Sami Heymann, Netaniel Rein, Marco Zurita, Revital Shechter, Zvi Israel, Michal Balberg, Mordekhay Medvedovsky, Guy Rosenthal

**Affiliations:** 1 The Faculty of Medicine, Hebrew University of Jerusalem, Jerusalem, Israel; 2 Department of Neurosurgery, Hadassah Medical Organization, Jerusalem, Israel; 3 Department of Neurology and Agnes Ginges Center for Human Neurogenetics, Hadassah Medical Organization, Jerusalem, Israel; 4 Neuro-Electro-Light Center (NELC), Hadassah Medical Organization and Holon Institute of Technology, Jerusalem, Israel; 5 Faculty of Electrical and Electronics Engineering, Holon Institute of Technology, Holon, Israel

**Keywords:** intracranial fNIRS (ifNIRS), optical anchor bolt (OAB), depth optrode (DO), stereotactic EEG (SEEG), swine model

## Abstract

**Introduction:**

Functional Near-Infrared Spectroscopy (fNIRS) is widely used to monitor cerebral hemodynamics, however, it is limited by shallow penetration depth and susceptibility to hemodynamic noise from the scalp. A novel intracranial fNIRS (ifNIRS) system, featuring depth optrodes (optode-electrodes) and optical anchor bolts (OABs), has been proposed to address these limitations. This study investigates the feasibility of ifNIRS in a swine model under controlled interventions.

**Methods:**

Three animals were implanted with ifNIRS. Each animal with three OABs, with depth optrodes (DO) inserted into two of the OABs. Hemodynamic changes were recorded using OAB-to-OAB (OAB-OAB) and DO-to-OAB (DO-OAB) channels. Two interventions were performed to generate hemodynamic changes: rapid infusion of hypotonic saline to induce cerebral edema and blood withdrawal. Postmortem assessment for tissue damage and hemorrhage was performed. Hemoglobin concentration changes were analyzed using the Beer-Lambert equation.

**Results:**

A decrease in total hemoglobin (tHb) levels during blood withdrawal was observed in all channel configurations that displayed relevant signals. During hypotonic saline infusion, variable patterns of tHb were observed. Postmortem findings showed minor extra-axial hemorrhages near OABs, but no intracerebral or heat-related injuries.

**Discussion:**

This study demonstrates the feasibility of the ifNIRS system in detecting hemodynamic changes *in vivo*. While technical refinements are needed, ifNIRS shows promise for improving cerebral hemodynamic monitoring and enhancing diagnostic accuracy in invasive monitoring of patients with epilepsy.

## 1 Introduction

For decades, functional Near Infrared Spectroscopy (fNIRS) has been utilized to monitor cerebral hemodynamic changes across various conditions and activities (1–3). This technique employs near-infrared (NIR) light emitters and detectors (in the wavelength range of 690–1,000 nm) to measure fluctuations in the local concentrations of oxyhemoglobin (HbO) and deoxyhemoglobin (HbR). These measurements act as indicators of brain activity, relying on the principle of neurovascular coupling (4, 5).

Over the last four decades, fNIRS technology has advanced significantly, evolving from single-channel devices to advanced multi-channel systems with over a hundred channels. This progress led the way to its application across numerous clinical and research disciplines, including Neurology, Psychiatry, Psychology, Intensive Care, and fundamental research (2, 6). These systems offer distinct advantages, such as cost-effectiveness, high temporal resolution, extended monitoring capabilities during daily activities, and reduced sensitivity to motion artifacts compared to fMRI (7, 8).

While these systems offer notable advantages, scalp-based fNIRS systems face certain challenges. Current scalp-based fNIRS systems have restricted penetration depth, reaching only the outer cortical layers (9) due to physical and safety constraints. Additionally, hemodynamic changes originating in the scalp cause noise that interferes with the detection of intracerebral signals (10). Motion artifacts caused by poor coupling between optodes and the skin further degrade the signal-to-noise ratio (SNR), and spatial resolution remains limited to roughly 1 cm (11).

In the past, several studies have demonstrated the capacity of fNIRS to identify hemoglobin variations linked to seizures (12–14) and interictal discharges (15). These hemodynamic changes have been observed to precede the clinical and electrographic onset of seizures (16), though their underlying mechanisms remain poorly understood. Another study combined fast optical signal imaging with electrocorticography (ECoG) in a rat model of epilepsy and demonstrated that optical changes in neural tissue can be detected before and during interictal epileptic spikes (17). Diffuse optical tomography (DOT) combined with EEG has shown seizure-related hemodynamic responses in a neonate with hypoxic-ischemic encephalopathy (18), highlighting its diagnostic potential, though its use in epilepsy remains limited. The ability to identify preictal changes, possibly improve localization accuracy and deepen insights into ictogenesis, all support the integration of fNIRS into the diagnostic pipeline for patients with epilepsy (PWE).

Integrating fNIRS into the diagnostic workup of PWE presents an additional potential advantage. For patients with drug-resistant epilepsy (DRE), stereotactic EEG (SEEG) surgery is often performed, involving the implantation of electrodes into the brain through small burr holes in the skull to achieve precise localization of the seizure onset zone (SOZ) (19, 20). However, the effectiveness of SEEG can be limited when depth electrodes are not placed in close proximity to the SOZ, due to a “tunnel vision” effect (21). Unlike SEEG, fNIRS is not constrained by this limitation, and incorporating an fNIRS sensor alongside SEEG could help address this issue, potentially improving the detection and localization of the SOZ.

**Figure 1:**
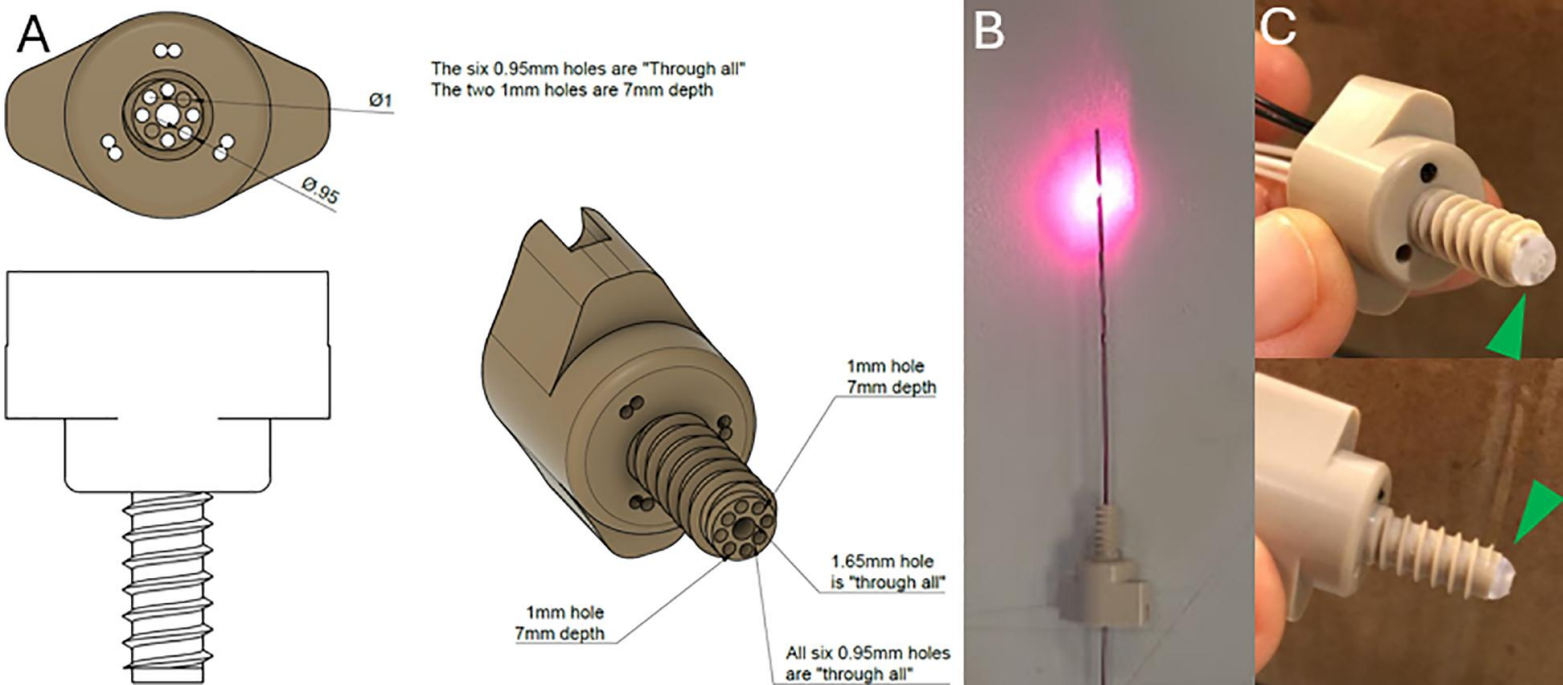
**(A)** Schematic diagram of the OAB made of polyether ether ketone (PEEK ®). **(B)** An implantable depth optrode. **(C)** two pictures of the OAB with the hemispherical hazy diffuser (green arrowheads) attached to the end of the bolt.

A recent study demonstrated, through Monte-Carlo (MC) simulation and phantom experiments, that many of the limitations associated with fNIRS could be addressed by employing an intracranial fNIRS (ifNIRS) system (22). This system consists of a depth optrode (which consists of a depth electrode and embedded optode) and an optical anchor bolt (OAB), which, in addition to housing a central cavity for the DO, features additional cavities for optodes positioned near the inner surface of the skull (Figure 1). The simulation of ifNIRS resulted in increased depth penetration, decreased susceptibility to hemodynamic noise from the scalp and demonstrated a potential increase of the axial detection range between OAB channels when compared to standard, scalp-based, fNIRS systems.

The important advantages demonstrated in the MC simulation led to the initiation of *in vivo* experiments to test the feasibility of ifNIRS. Early observations from the *in vivo* study were described in a supplementary to the aforementioned study (22). A potential challenge in the *in vivo* application of the ifNIRS system might be the presence of a small hemorrhage or a blood vessel, even just a few millimeters in diameter, located near an optode. Such an occurrence could obstruct the signal and render the system ineffective (23).

Herein we report the results from an *in vivo* study demonstrating the feasibility and practicality of ifNIRS towards its integration into a combined SEEG-ifNIRS system that will overcome the limitations of both scalp fNIRS and SEEG.

## 2 Methods

In order to assess the feasibility of implanted fNIRS (ifNIRS) *in vivo*, we performed a terminal experiment under general anesthesia after receiving approval from the institutional ethics committee of the Hebrew University (MD-23-17338-3). The study was performed at the animal surgical facility of the Faculty of Medicine, Hebrew University of Jerusalem.

### 2.1 Preparations

Three adult female swine, weighing between 31 and 40 Kg, were studied. The animals were housed in the animal care facility for 5–7 days before the procedure, with free access to food and water until 12 h before anesthesia induction. Premedication was administered via intramuscular injection of ketamine (20 mg/kg) and xylazine (2 mg/kg), followed by endotracheal intubation. Anesthesia was maintained with inhaled isoflurane using a mechanical ventilator (Narkomed 2B, Pennsylvania, USA) under controlled ventilation. A venous line was established for intravenous fluid administration, and a femoral arterial line was placed to facilitate arterial blood sampling. Throughout the procedure, arterial blood pressure, heart rate, blood oxygen saturation, and body temperature were continuously monitored and recorded.

### 2.2 Surgical procedure

The swine were positioned in the prone position. A lateral skull x-ray with radiopaque markers was performed to determine optimal burr-hole sites and estimate bone thickness. A midline linear skin incision was made, and a self-retaining retractor was used to expose the skull surface.

Four burr holes (5 mm in diameter) were drilled using a high-speed electric drill. Two holes were placed anterior to the coronal suture on either side of the sagittal suture, 2.5 cm apart, and two additional holes were positioned 2.5 cm posterior to the first set.

Three of the burr holes were drilled to full depth, exposing the dura mater, which was subsequently punctured using a 20-gauge needle. The fourth burr hole, designated for the OAB through which emitting DO were not inserted, was drilled to partial thickness, without penetrating the tabula interna. Bony hemostasis was achieved using bone wax which is a hemostatic agent used in neurosurgery with minimal complications (24, 25).

An intraparenchymal ICP monitor (Spiegelberg, Hamburg, Germany) was inserted through one of the burr holes, to a depth of 1 cm. Bone wax was used to seal the burr hole and secure the probe in place. ICP was continuously recorded using a critical care monitor (Datex-Ohmeda, Helsinki, Finland).

For the remaining three burr holes, a screw thread tap was used to create helical grooves within the bone. Three OABs were screwed in until a firm grip was achieved. Optical fibers, coupled to sources and detectors were inserted into the designated apertures of the OABs, while DOs containing NIR emitters were inserted through the OABs into the brain parenchyma.

### 2.3 Intervention protocol

The first intervention involved the implementation of a model of induced intracellular cerebral edema by rapid infusion of hypotonic saline (6l of 0.18% NaCl over 120–150 min). This model of water intoxication, previously described in detail in our earlier work, leads to a gradual and sustained increase in ICP as cerebral edema progresses (26).

The second intervention consisted of blood withdrawal, with the withdrawal of 2.5l of blood through an arterial cannula.

Finally, potassium chloride (KCl) was administered as part of the terminal procedure, with continuous monitoring of cardiac activity until cessation.

Postmortem dissection of the skull and brain was performed to assess the presence of hemorrhages, heat injury, or other forms of tissue damage.

### 2.4 Intracranial fNIRS (ifNIRS) system

#### 2.4.1 Optical anchor bolt (OAB) structure

The bolt was manufactured from polyether ether ketone **(**PEEK©) to allow fixation through the bone. This material was selected due to its relative strength and being MRI safe and biocompatible material used in cranioplasty (27). It consists of a head and a shank with through-holes with a diameter of 0.95–2 mm. Some optodes can be inserted through the head of the bolt, providing non-invasive readings through the scalp, and some optodes can be positioned through the shank inside the skull. The length of the shank was determined according to the mean skull thickness of a 40 kg swine. We note that the bolt terminates inside the skull and does not penetrate the dura. This reduces the likelihood of bleeding and obstruction of the fiber tips. The end of the bolt is covered by hemispherical hazy optical diffuser, made from BioMed Clear Resin (Formlabs, Somerville, MA, USA), also containing a through-hole for the DO to be inserted through. The DO can be inserted through a 1.65 mm opening in the center of the bolt. Pictures of the OAB and DO and a schematic diagram of the OAB are presented in Figure 1.

#### 2.4.2 Depth optrode (DO)

A standard SEEG electrode (Sensomedical Ltd., Israel) was modified to include optical fibers ending in a diffusive ring (dimensions: height-3 mm, radius-0.8 mm). The diffusive ring was placed about 1.5 cm from the distal tip of the electrode, between the electrical contacts. The ring was made from scattering Thermoplastic Polyurethane (TPU). Before surgically inserting the electrodes, the optical fibers (multimode fiber—62.5/125 μm) were inserted into the SEEG electrode via thin Teflon tubing.

#### 2.4.3 fNIRS system

A versatile research-grade fNIRS system was built in our lab allowing various arrangements of emitters and detectors, and control of the sequence of operation of the emitters. The system consisted of two pairs of lasers (IPS, USA), at two wavelengths—785 nm and 830 nm, with an average continuous power of 80 mW and 8 Avalanche photodetectors (C5460 by Hamamatsu Inc., Japan). The output of the lasers was split into 8 sources, using optical switches (Lightwaves Link, China). The optical switches were controlled using a multi-purpose digital controller (Measurement Computing Corporation, USA), and the analog to digital conversion of the detected light signals was performed using the same system. Each source emitted either 785 nm or 830 nm, according to a predetermined schedule. The system was operated using a maximal duty cycle of 1:16. The sources were coupled to a multimode (MM) fiber (62.5/125 μm) which was then inserted into the OAB or a DO. In the case of inserting the fiber to the OAB, the distance between the tip of the fiber and the tissue was 2 mm. The resulting power density was 250 mW/cm^2^, which is below the maximum permissible exposure limit (MPE) for skin (ANZI.2136.1). In the case of the DO the fiber was inserted into the DO up to the diffusive ring with an area of 0.15 cm^2^ and power density of up to 67 mW/cm^2^. The detection was done with MM fibers varying from one 400 μm core fiber to a bundle of eight 200 μm core fibers. The detection fibers were connected to customized avalanche photodiodes via a SMA connector in free space. Emitters in the DOs and OABs and detectors in the OABs were positioned to create OAB-to-OAB (OAB-OAB) channels and DO-to-OAB (DO-OAB) channels. The light was measured continuously at a sampling rate of 4–8 Hz and then averaged over 10 min periods.

### 2.5 Signal analysis

The change in the concentrations of HbO and HbR from the measured intensity of near-infrared light at two wavelengths was calculated using the Beer–Lambert law (28).

The Beer–Lambert law relates absorbance to concentration as:

$$\Delta A_{\lambda }=-\ln\;\left(\frac{I_{\lambda }}{I_{0,\lambda }}\right)$$ (1)

where ΔA*_λ_* is the relative absorbance at wavelength *λ*, I_0,*λ*_ is the average detected intensity during the first 10 min and I*_λ_* is the detected intensity.

For two chromophores (HbO2 and Hb) at two wavelengths

$$\Delta A_{\lambda_{1}}=L^{\prime }\;(\varepsilon_{HbO}(\lambda_{1})[HbO]+\varepsilon_{HbR}(\lambda_{1})[HbR])$$ (2)

$$\Delta A_{\lambda_{2}}=L^{\prime }(\varepsilon_{HbO}(\lambda_{2})[HbO]+\varepsilon_{HbR}(\lambda_{2})[HbR])$$ (3)

where L′ is the effective optical pathlength (L′ = L × DPF, where L is the distance between the emitter and the detector and DPF is the differential pathlength factor that accounts for scattering) and *ε* are the extinction coefficients for each chromophore extracted from (29), for *λ*1 = 785 nm and *λ*2 = 830 nm.

The change in the concentration of each chromophore is given by:

$$\Delta [HbO]=\frac{\Delta A_{\lambda_{1}}\varepsilon_{HbR}(\lambda_{2})-\Delta A_{\lambda_{2}}\varepsilon_{HbR}(\lambda_{1})}{L^{\prime }(\varepsilon_{HbO}(\lambda_{1})\varepsilon_{HbR}(\lambda_{2})-\varepsilon_{HbO}(\lambda_{2})\varepsilon_{HbR}(\lambda_{1}))}$$ (4)

$$\Delta [HbR]=\frac{\Delta A_{\lambda_{2}}\varepsilon_{HbO}(\lambda_{1})-\Delta A_{\lambda_{1}}\varepsilon_{HbO}(\lambda_{2})}{L^{\prime }(\varepsilon_{HbO}(\lambda_{1})\varepsilon_{HbR}(\lambda_{2})-\varepsilon_{HbO}(\lambda_{2})\varepsilon_{HbR}(\lambda_{1}))}$$ (5)

Following the averaging of the signal over 10-minute intervals, the relative error for each averaged data point was determined by evaluating the contribution of measurement noise in the detected intensity (±0.01 V) to the computed total hemoglobin concentration. Given a sampling rate of approximately 4 Hz, each time point represented an average of −2,400 individual measurements, yielding a relatively low uncertainty of approximately 0.6% in the calculated values.

## 3 Results

**Figure 2:**
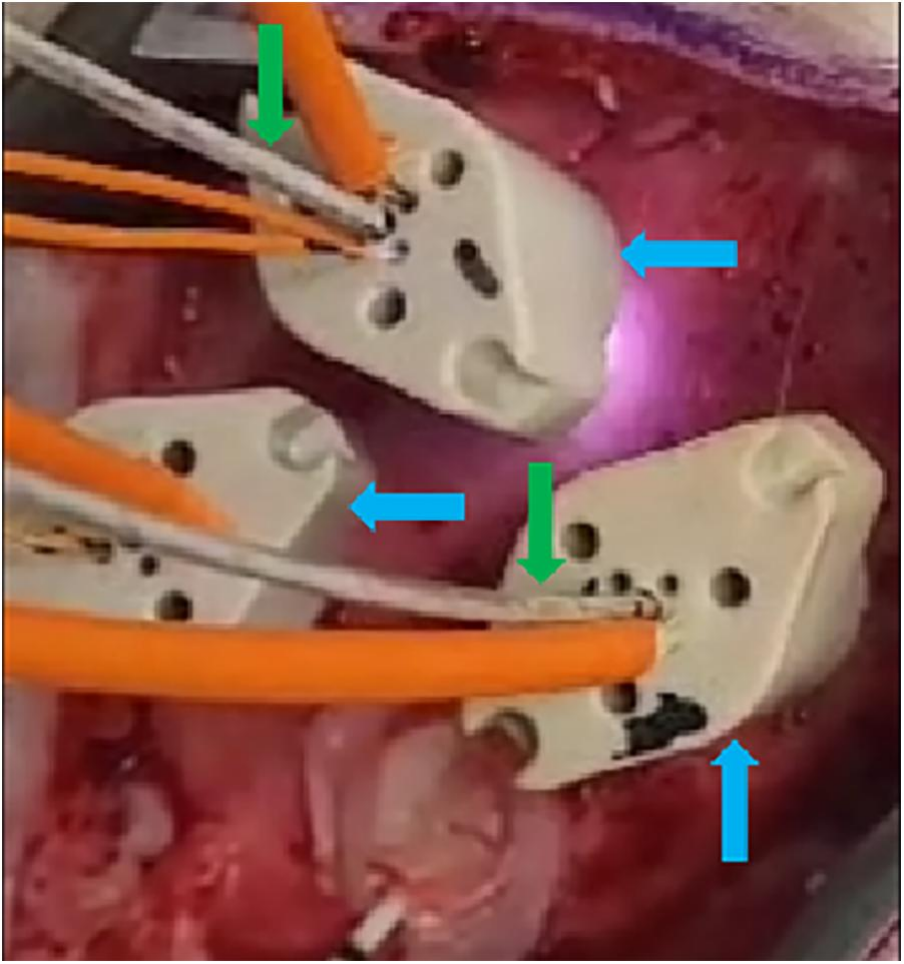
Three OABs (blue arrows) implanted into the exposed skull through burr holes (not visible) of one of the animals. Two of the OABs have depth optrodes (green arrows) implanted through them, and all of them have intraosseous optodes inserted into the designated apertures (orange wires).

**Figure 3:**
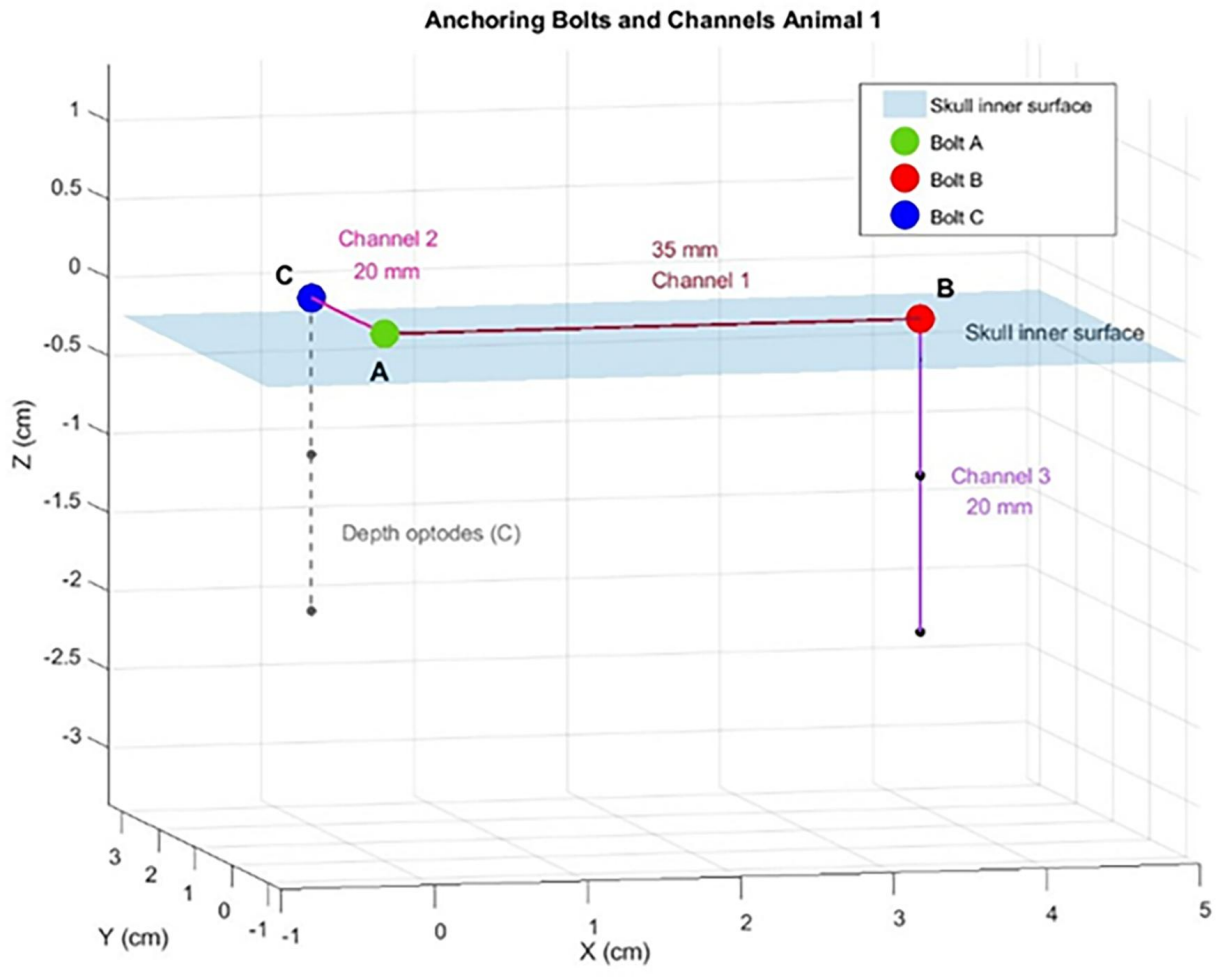
A schematic plotting of the position of the optical anchor bolts (OABs), depth optrodes (DO) and the channels from the swine #1. A,B,C represent the location of the three OABs on a planar surface representing the skull inner surface (blue shaded plane). The distances between the different channels are marked on a line connecting the OABs.

Each animal was implanted with three OABs, with emitting DOs inserted through two of them (Figure 2). Since each emitter could potentially pair with each detector, the total number of possible channels per animal was the product of emitters and detectors. A schematic representation of the positioning of the tip of the three OABs and the two depth optrodes relative to the inner surface of the skull of the first animal can be seen in Figure 3. In the first swine—used here as a representative example—three emitters (one in each optrode and one in bolt A) and three detectors (one per OAB) yielded nine potential channels. Of these, six were excluded: four due to signal below threshold of 0.01 V during the full duration of the experiment for either wavelength between depth optrodes and non-matching OABs, one due to similar below threshold signal between a DO and its corresponding OAB, and one due to constant signal saturation (above 4.1 V) for at least one of the wavelengths when emitter and detector were on the same OAB. This left three usable channels. Using similar channel exclusion criteria for minimum threshold or signal saturation, the second and third animals had four and five usable channels, respectively.

In total, following this exclusion process, nine OAB–OAB channels and three DO–OAB channels were deemed usable (Table 1). Inter-OAB distances ranged from 20–35 mm, and DOs were positioned 15–20 mm below the skull's inner surface.

**Table 1:** List of channels from all swine. OAB-OAB—channel connecting two optical anchor bolts (OAB). DO-OAB—Depth optrode to OAB channel.

Number of animal	Type of channel	Emitter-detector separation (mm)
1	OAB-OAB	35
1	OAB-OAB	20
1	DO-OAB	20
2	OAB-OAB	20
2	OAB-OAB	24
2	DO-OAB	20
2^a^	DO-OAB	20
3	DO-OAB	20
3^a^	OAB-OAB	22
3	OAB-OAB	20
3	OAB-OAB	20
3	OAB-OAB	24

a channel not usable due to signal saturation or signal falling below noise threshold during blood.

**Figure 4:**
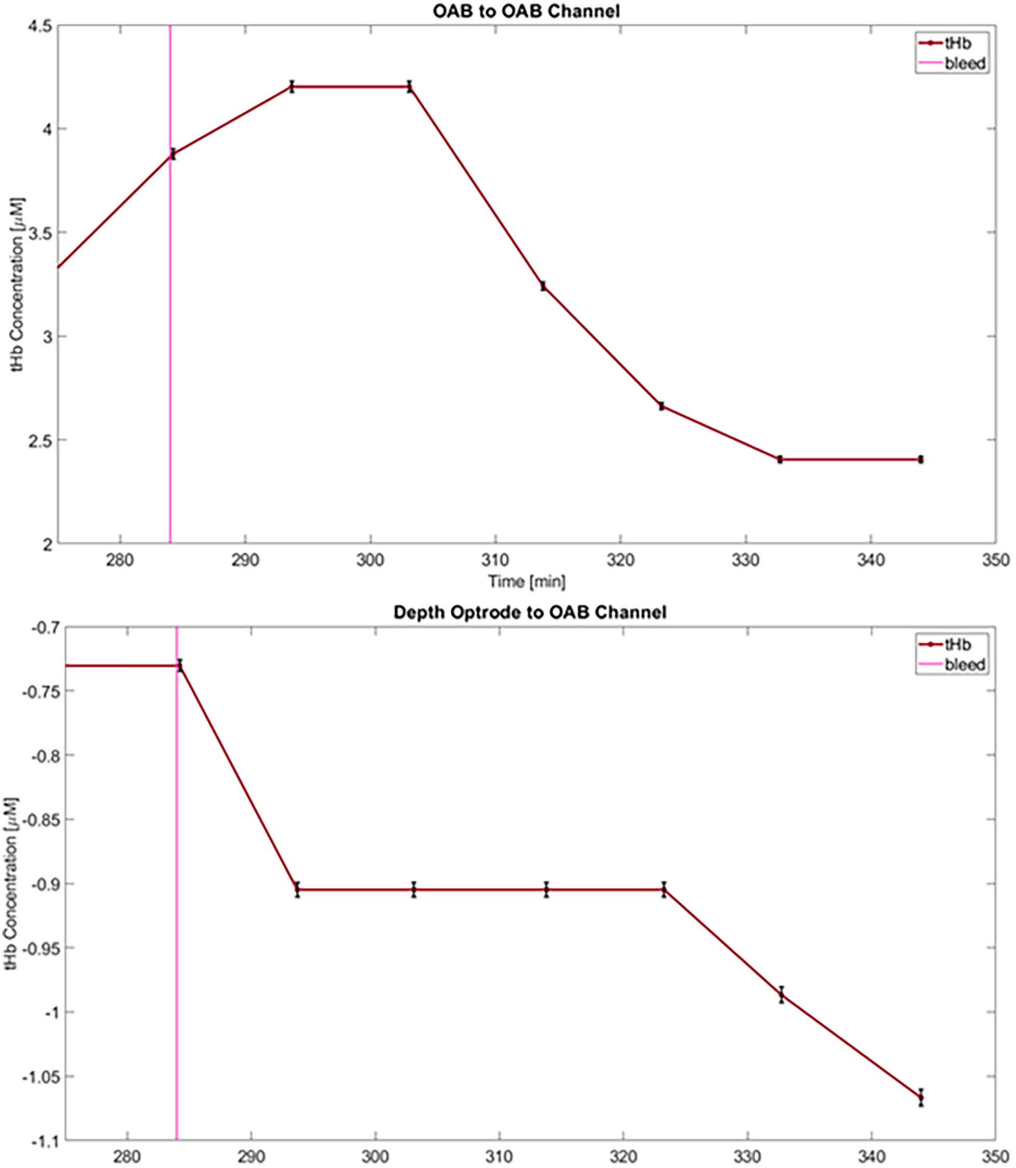
Changes in total hemoglobin concentration (tHb) from baseline following blood-withdrawal onset (at 284 min—pink vertical line) from an OAB-to-OAB channel (top) and a depth-optrode-to-OAB channel (bottom) from the first animals. Data points represent measurements averaged over 10 min, and error bars for each data point are marked (black) representing an uncertainty of approximately 0.6%.

Ten of the 12 channels that provided reliable signals demonstrated an average decrease in tHb of −1.09 μM (range 0.336–2.58 μM) during the blood-withdrawal phase (Figure 4); these findings are considered robust given the low measurement uncertainty (−0.6%). Various trends were detected in the ratio of HbO and HbR (see Supplementary Figure S1). The measurement in the two remaining channels during the blood-withdrawal phase was impaired due to either signal saturation in one of the sources or minimal signal in the other.

**Figure 5:**
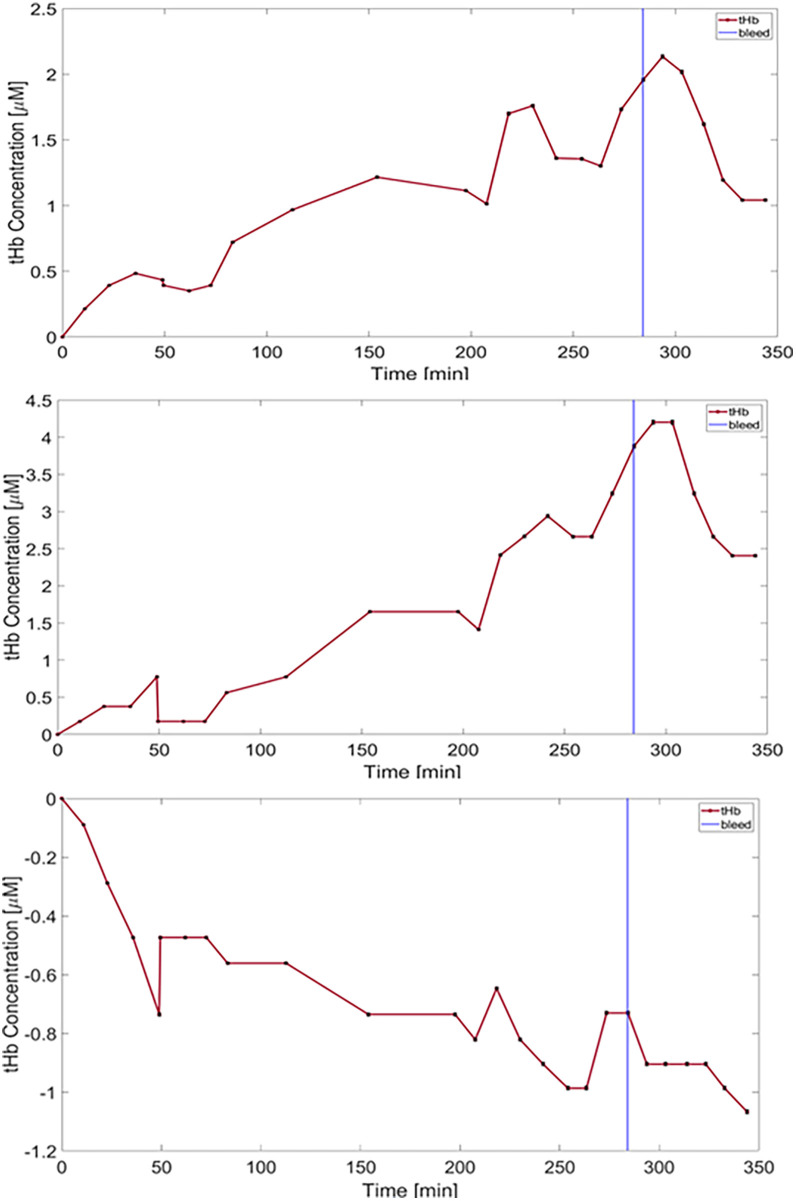
Plots of total hemoglobin concentration (tHb) over time are shown. Error bars are shown in black representing an uncertainty of approximately 0.6%. The top two top plots represent data recorded from the two OAB-to-OAB channels of the first animal, while the bottom plot corresponds to DO-to-OAB channel of the same animal. Hypotonic saline was infused from the start of the experiment, followed by blood withdrawal (vertical blue line -284 min).

During the infusion of hypotonic saline, different trends were apparent. In the first animal an increase in tHb in the OAB-OAB channels and a decrease in the DO-OAB channels was measured (Figure 5).

In the second animal different trends were seen during the rapid infusion phase.

In the third animal a gradual increase in tHb was seen in all channels from the onset of the rapid infusion phase. In the DO-OAB channel a slow decrease in tHb beginning −40 min prior to the blood-withdrawal phase was detected, which continued through the blood-withdrawal phase, while the OAB-to-OAB channels showed a decrease in tHb only in the blood-withdrawal phase as stated above.

Postmortem examinations revealed two minor extra-axial hemorrhages beneath the positions of the OABs, yet signal variations were still detected despite the presence of bleeding. No intracerebral hemorrhages or heat-related injuries were identified in the brains of the animals during postmortem analysis.

## 4 Discussion

In this study, we demonstrated the implementation of the ifNIRS system in an *in vivo* experiment conducted on a swine animal model. Changes in hemoglobin concentration were successfully measured, correlating with interventions across both OAB-OAB and DO-OAB channels, thereby establishing the feasibility of ifNIRS monitoring. Postmortem examination revealed no evidence of heat-related injuries or intracerebral bleeding, with only minor extra-axial hemorrhages observed.

Rapid infusion of hypotonic saline and blood withdrawal were chosen as interventions due to their simplicity and the familiarity of our team with the former. The study aimed to demonstrate the capability of ifNIRS to monitor *in vivo* changes in Hb concentration, with this model effectively controlling the hemodynamic state of the brain. Although swine epilepsy models exist (30, 31), the simpler approach was selected, as it met the objectives of the study without introducing additional complexity.

During the experiments, the system proved to be reliable, with no large fluctuations or other malfunctions. A low signal or detector saturation during the blood-withdrawal phase of the recording limited the usability of data from two out of 12 channels during the experiment. Based on these findings, adjustments to the system are planned to enhance its applicability across a broader range of conditions. While the animal experiment was required to demonstrate the applicability of the system, these technical challenges discovered during the animal trials highlight the critical role of *in vivo* experimentation in refining the design prior to human trials.

The calculated decrease in tHb levels during the final blood withdrawal demonstrates the future utility of the ifNIRS system. The varying changes in measured tHb, HbO and HbR during the rapid infusion phase raise interesting questions regarding the hemodynamic changes that may occur during cerebral edema. Several previous studies in both animals and humans described the macrovascular and microvascular changes brought on by cerebral edema (32–35). In addition to these mechanisms, the positioning of the components of the ifNIRS system provides another possible explanation for the variability in measurements. It could be hypothesized that the progression of cerebral edema reduces the proportion of cerebrospinal fluid (CSF) within the cranial vault as the cortex becomes compressed against the skull. This may effectively eliminate the CSF layer, which has low scatter and absorption coefficients (36, 37), and replaces it with brain tissue, characterized by higher optical coefficients. This substitution leads to a decrease in measured NIR intensity and an increase in calculated Hb levels. Such an effect is expected to be more pronounced in OAB-OAB channels, while in DO-OAB channels, it may be counteracted by the longer path of NIR light through the edematous brain. This pathway is influenced by compressed blood vessels and blood dilution caused by the rapid infusion of hypotonic saline. While this theory aligns with the findings observed in the first animal, further targeted studies are required to validate it. If corroborated, this mechanism could contribute to the development of a method for monitoring patients with cerebral edema. It may provide an early warning of an impending rapid increase in intracranial pressure (ICP) by detecting the brain's compression against the skull before the significant loss of intracranial compliance. Whether resulting from cerebral edema, vasospasm, or shifts in the relative volumes of intracranial compartments, or any combination of these mechanisms, the variable hemodynamic responses observed during rapid saline infusion warrant further investigation to elucidate the underlying physiological mechanisms.

Several additional steps are required to further develop the ifNIRS system before initiating human *in vivo* experiments and eventual clinical applications. Replicating the current experiment in a larger cohort of animals, incorporating temperature monitoring near the emitters, while continuing to perform postmortem dissections to assess potential tissue damage caused by emitters positioned deep within the brain are essential next steps. Regarding safety, it is noteworthy that recently reported pre-ictal hemodynamic changes detected by fNIRS in patients occur at very low frequencies (−0.002–0.02 Hz) (38), necessitating a correspondingly low sampling rate. This implies reduced energy use and, consequently, a lower risk of thermal injury to brain tissue. In addition to safety considerations, The observed variability in hemodynamic responses underscores the necessity for additional animal studies to deepen our understanding of fNIRS-detected hemodynamic changes originating from deep brain regions. Given that peri-ictal *in vivo* fNIRS measurements from such regions remain largely unexplored, an initial phase of data interpretation and methodological refinement should be anticipated in early human trials before any clinically meaningful conclusions can be confidently drawn, even following extensive preclinical investigations.

The postmortem analysis revealed only two minor extra-axial hemorrhages with no visible tissue damage or intracerebral hemorrhage. The consistent detection of intervention-related signals by the ifNIRS system, even in the presence of small extra-axial hemorrhages beneath the OAB, demonstrates a robustness to hemorrhage effects greater than initially anticipated. The ability of the system to maintain detection despite hemorrhage may be facilitated by the hazy, nearly hemispherical optical diffuser mounted on the inner edge of the OAB, which expands the angular field for both incoming and outgoing light. The absence of additional tissue damage or intracerebral hemorrhage suggests the relative safety of the system, although the small number of animals and the limited total of OABs and DOs studied constrain the strength of this conclusion.

This study has several limitations. A small number of animals was used, resulting in a limited dataset. This sample size was deliberately selected to provide sufficient data to demonstrate the feasibility of ifNIRS measurements while minimizing animal use, in accordance with the principle of reduction in animal experimentation (39). Data usability was constrained in some channels due to detector saturation and signal absence, suggesting the need for adjustments to the system. Although postmortem analysis did not reveal evidence of tissue damage, intracerebral temperature changes were not measured.

## 5 Conclusion

This study demonstrated the feasibility of the ifNIRS system in a swine model, with measured hemodynamic changes correlating with experimental interventions. Technical challenges, including signal inconsistencies, provide valuable insights for system refinement. This work represents a critical step toward the integration of ifNIRS into clinical practice.

## Data Availability

The raw data supporting the conclusions of this article will be made available by the authors, without undue reservation.
