# Supplementary material for: Intracranial functional near-infrared spectroscopy: an animal feasibility study
Source: Front Med Technol. 2025 Nov 27;7:1692573. doi: 10.3389/fmedt.2025.1692573 (PMC12695811; doi:10.3389/fmedt.2025.1692573)
Supplement: Supplementary file 1 [file Datasheet1.docx]

Supplementary Material

# Supplementary Figures
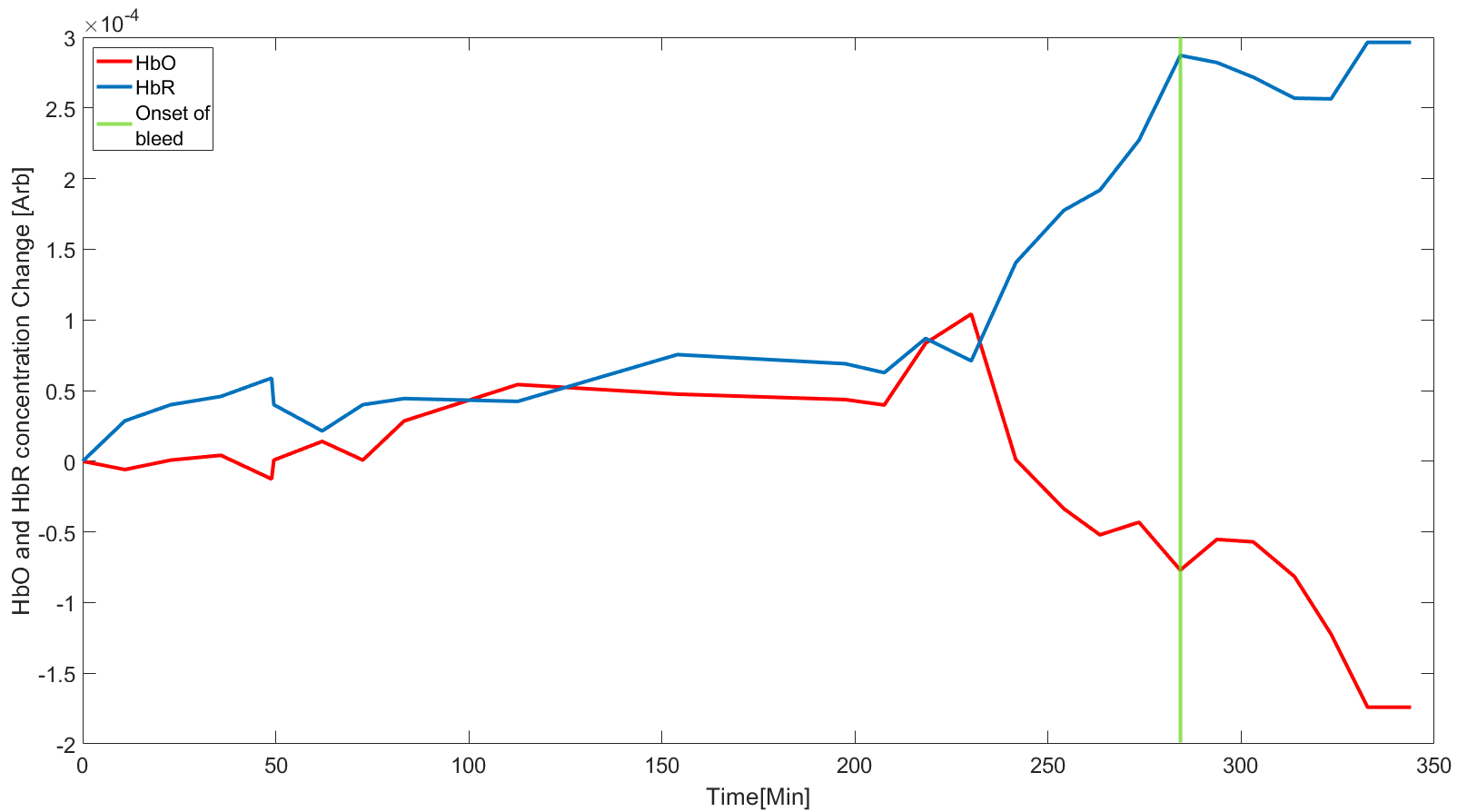

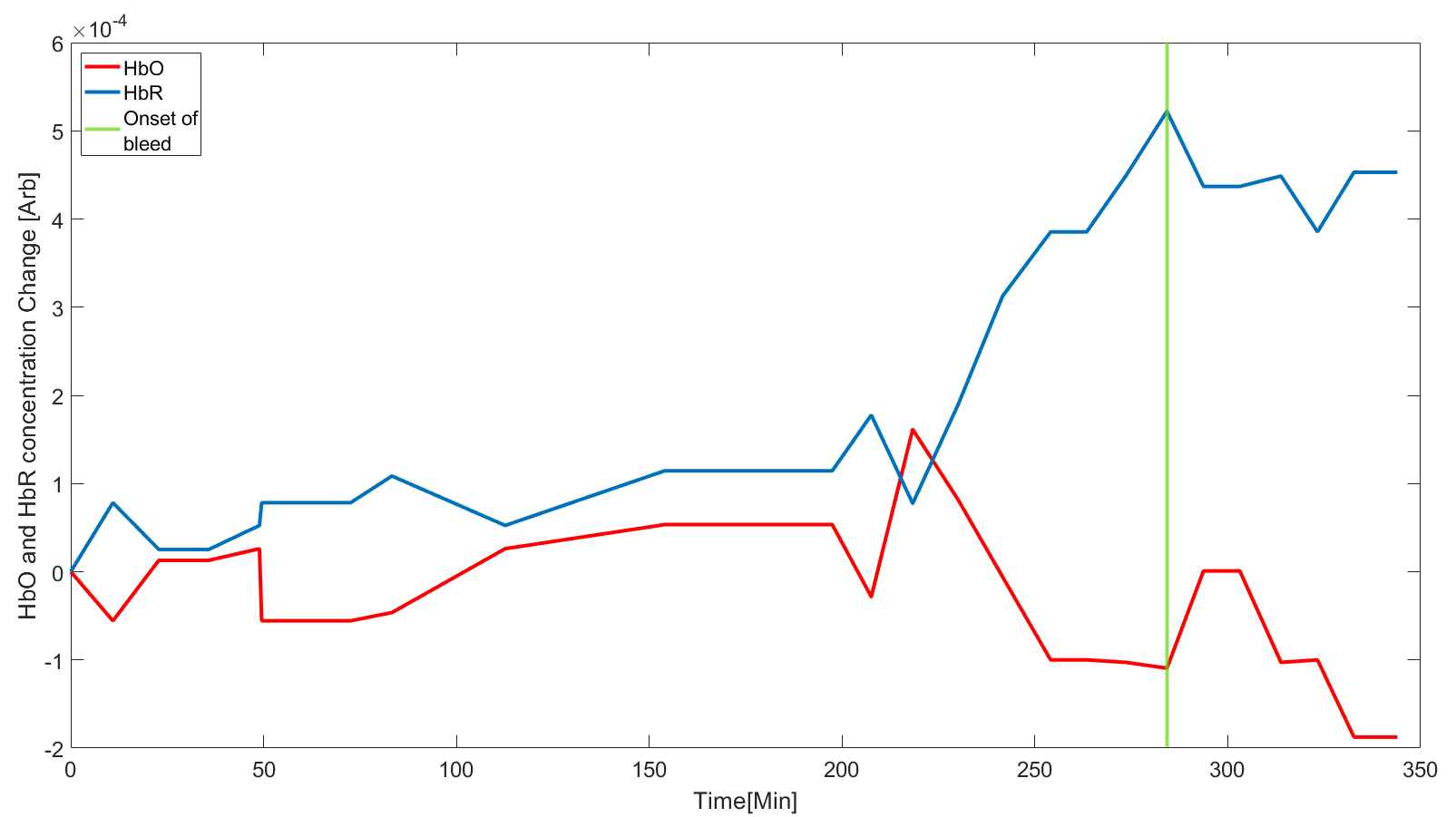

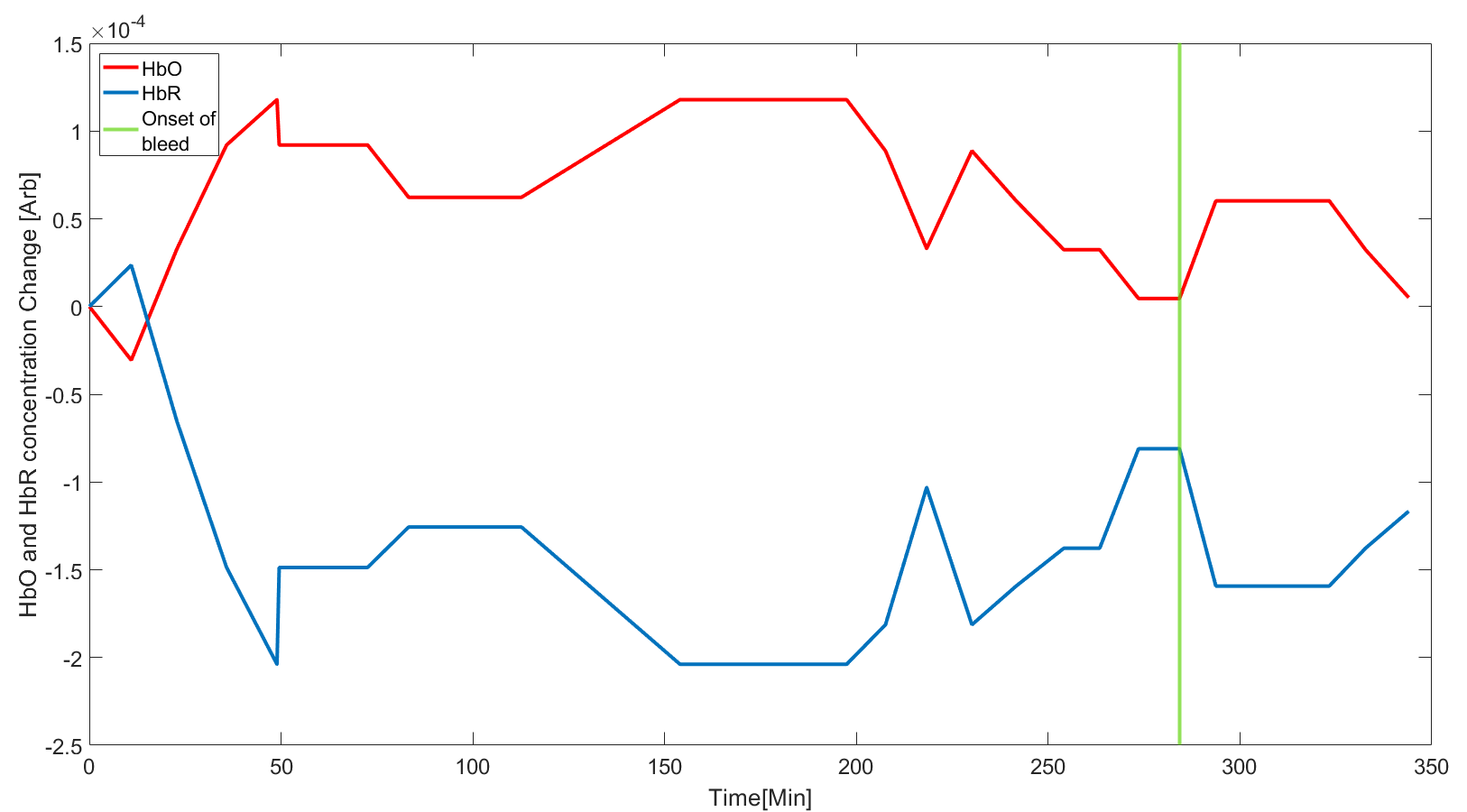


**Supplementary Figure 1** - Plots of changes in oxyhemoglobin (HbO, red line) and deoxyhemoglobin (HbR, blue line) levels over time from the first animal are shown. The two upper plots display data from optical anchor bolt (OAB)–to–OAB channels, while the lower plot shows depth optrode (DO)–to–OAB data, corresponding to the total hemoglobin plots presented in the main text (Figure 5). Hypotonic saline was infused from the start of the experiment, followed by blood withdrawal (vertical green line).

Similar trends can be observed in both OAB-to-OAB channels, with relatively stable HbO and HbR concentrations during the first ~200 minutes. In contrast, the DO-to-OAB channel showed an early increase in HbO accompanied by a decrease in HbR. Around 220 minutes, all channels demonstrated a decrease in HbO and an increase in HbR, with these changes slowing or ceasing once blood withdrawal began. The simultaneous onset of change across all three channels at ~220 minutes suggests a relationship between a physiological event, such as arrested perfusion due to cerebral edema, and alterations in calculated hemoglobin levels. The differing HbO and HbR trends observed in OAB-to-OAB versus DO-to-OAB channels may be explained by the distinct effects of cerebral edema on the layer in the path between optodes in each channel type, as discussed in the main text, although further experimentation is required to confirm this.
